# Reply to “Letter to the Editor: Consistency Among Musculoskeletal Models: *Caveat Utilitor*”

**DOI:** 10.1007/s10439-014-1152-z

**Published:** 2014-11-04

**Authors:** David W. Wagner, Vahagn Stepanyan, James M. Shippen, Matthew S. DeMers, Robin S. Gibbons, Brian J. Andrews, Graham H. Creasey, Gary S. Beaupre

**Affiliations:** 1 Center for Tissue Regeneration, Repair, and Restoration, VA Palo Alto Health Care System, 3801 Miranda Ave, Palo Alto, CA 94304 USA; 2 Department of Mechanical Engineering, Stanford University, Stanford, CA USA; 3 Industrial Design, Coventry University, Worcestershire, UK; 4 School of Sport and Education, Brunel University, Middlesex, UK; 5 Nuffield Department of Surgical Sciences, Oxford University, Oxford, UK; 6 Spinal Cord Injury Service, VA Palo Alto Health Care System, Palo Alto, CA USA

**Keywords:** Musculoskeletal models, Muscle moment arm, Joint contact force, Muscle recruitment, Musculoskeletal simulation, Knee flexion

To the Editor,

We would like to thank Dr. Modenese and colleagues for taking the time to discuss our article “Consistency Among Musculoskeletal Models: Caveat Utilitor”.^6^ We would also like to thank those same researchers for making the model developed by their group, the London Lower Limb Model (LLLM),^5^ available to the musculoskeletal modeling community. After reviewing their analysis, we do agree with the LLLM researchers that the large discrepancy in quadriceps muscle recruitment (Fig. 4 in our original article) produced from their model as compared with the other models that were analyzed was primarily related to the fact that “neither contraction dynamics nor force–length–velocity relationships were implemented for the muscle actuators”^5^ in their model.

The intent of our original analysis was not to misrepresent the LLLM model. The intent was to employ the models in canonical analyses shared by their respective software and advise future model users. As the purpose of the original study was to “compare eight models from three software packages and evaluate differences in quadriceps moment arms, predicted muscle forces, and predicted tibiofemoral contact forces…” we selected standardized methods and analysis techniques for all models and all software packages. More specifically, we selected the methods most likely to be adopted by future users of the models and software. For example, in OpenSim, the standardized methods account for muscle force–length relationships by default and a biomechanist selecting a model today is most likely to adopt these standardized, default methods. Additionally, it should be noted that the LLLM model does specify the relevant muscle fiber length and tendon length parameters, regardless of whether those parameters are valid. We regret that the muscle recruitment force results from the LLLM were produced from an analysis that was not originally intended to be used with the LLLM and that the results were interpreted as a potential modeling issue. The remaining models implemented in the same OpemSim modeling software^2^ did not have the same issue as the LLLM model, nor did the model constructed in the AnyBody Modeling System^1^ that was based on the same Klein-Horsman anatomical data set.^3^ We agree with the LLLM researchers that, “attempting to contact the developers of a model to clarify uncertainties is … good practice”. The necessity to do so, however, may not always be obvious to the users, as was the situation with our use of their model; at the time we believed we were using the model as intended by the developers.

The discrepancy raised by the LLLM researchers suggests that there remain several substantial hurdles for musculoskeletal modeling to be widely accepted and adopted as an everyday engineering tool. We thank the LLLM researchers for clarifying the limitations of their model and for providing guidance on how to use their model to more closely match other currently available musculoskeletal models. We believe that this healthy discussion is critical for the growth and acceptance of musculoskeletal modeling as an engineering discipline, a direction we hope the engineering community is heading toward. We believe it is an important and not often discussed topic, and one that we briefly summarize in the “Future Research” section of the original article: “For musculoskeletal simulation to be widely adopted and incorporated as an engineering discipline, verification and validation methods that are common to other computer aided engineering modalities must be more widely incorporated.^4^ Consistent results between generic musculoskeletal models is one step toward accomplishing that goal such that a biomechanical analysis performed by one investigator at one location with one piece of software produces the same reliable and repeatable results as the same analysis performed by another individual, at another location, with another musculoskeletal simulation software package.”
